# Supplementary material for: Genomic SELEX Reveals Pervasive Role of the Flagella Master Regulator FlhDC in Carbon Metabolism
Source: Int J Mol Sci. 2023 Feb 12;24(4):3696. doi: 10.3390/ijms24043696 (PMC9962212; doi:10.3390/ijms24043696)
Supplement: Supplementary file 1 [file ijms-24-03696-s001.zip › ijms-2128467-supplementary.pdf]

Table S1. Primers used in this study.

(A) For gel shift assay

name	sequence
fliA-F	GGTACCGCAGCAGGTTCTGTCTCTGCT
fliA-R	CTTCGTGACGCACCAGCGGGAC
flgA/flgB-F	GATCGCCACGCTACGTTTTATTATCAGCAT
flgA/flgB-R	GGCGGCGTCGAGCTTATCGAGCAT
ptsH-F	GCCGATCTCTTCACTGAGAAAGAATTGCAA
ptsH-R	AGCGGTAATGGTAACTTCTTGCTGGAACAT
pfkA-F	TTCCGGGATCGGATGTAATTATCCATCAGG
pfkA-R	CATCACCGCCGCTTGTCAACACA
adhE/ychE-F	TTTACACGCTCTACGAGTGCGTTAAGTTCA
adhE/ychE-R	GGTCTGAATCACGGTTAGCTCCGAAGC
aceB-F	CTACGGCACATGAATCCAACGCTGGATTAA
aceB-R	TTCATCGGTTGTTGTTGCCTGTTCAGTCAT
fruB/setB-F	ATGGATGTCCTGTACGGATAACTGGAACAT
fruB/setB-R	GGAGACTGCGGGGGAGTTATGCAT
epd-F	CTGAAGAAGGCCGGTATCACTTCACAAGCT
epd-R	AAGCCATTTATCGCTACGCGTACGGTCAT
iclR/metH-F	GTTTCGCGGGAATGGGTGCGACCAT
iclR/metH-R	CACGCAGTTGTTCCACTTTGCTGCTCACAC
yqjI/yqjH-F	GCGGGTAGCGGGGGGTGTTATTCAT
yqjI/yqjH-R	TTTACAACACCCTTCGTGATGATGGCTCAT
purH/rrsE-F	CGTTTGCGCAACGCTCGCG
purH/rrsE-R	CTTCGAGTGCCCACACAGATTGTCTG

(B) For Northern blot analysis

fliA-F	TGCGTCACGAAGCATTGCGCCTGC
fliA-R	GGCTGTTATTGGTGTCGAGCAACATTTGGCGA
flgB-F	ATGCTCGATAAGCTCGACGCCGCCTTACG
flgB-R	TGGTATTGCAGGCTGTTATCGGCAAACTGG
ptsH-F	ATGTTCCAGCAAGAAGTTACCATTAC
ptsH-R	TTACTCGAGTTCCGCCATCAGTTTAA
fruB-F	AGGCCGGAGACAAAGAAGAGGCGATTCG
fruB-R	CTTCTGCTGTTGTTGCTGACTTCAGTTGTTCAGC
setB-F	TGACCTGACCTCGACGGCGTTTTTAATCGTTG
setB-R	CAAGGGCAAACATTTGCGGGTTAGCGGT
pfkA-F	AAATCGGTGTGTTGACAAGCGGCGGTGATG
pfkA-R	GCACGGGAAGCCCATTTCGGTCAGACG
epd-F	GGTCGCATCGGGCGTAATGTGGTTCGT
epd-R	TCGAGATCGTTACTGCCAGGATGTGAAAAGAGCAC
adhE-F	GTAGACAAAATCTTCCGCGCCGCCGCTC
adhE-R	CGGGTGCGGGGAGAAGATAATGGCGTTACG
aceB-F	GCGGTAGAATTTCTGACTGAGCTGGTGACGC
aceB-R	GTGTAACTGATGGTGCCGTTAACCGCATCACG

iclR-F	ACTGGACAGGTTCAGTCTTTAACGCGTGGC
iclR-R	GCGTACACTGTACCTGGTCGATAATAATCGCTTCG
metH-F	GGTGCTGGACGGCGGTATGGGCA
metH-R	CGAGAACACCGGCAACGTAGCGCGG
yqjI-F	CATGAAGGCCAGCCACGCCATGAGG
yqjI-R	CACTGCGCGCCTTGTTCGGTCAG
yqjH-F	GTTCGCAATGATCTGCGCTTCCGTGAACTG
yqjH-R	GCGCGGACCTGCCACCGTAAGT

DNA band patterns of genomic DNA fragments on PAGE

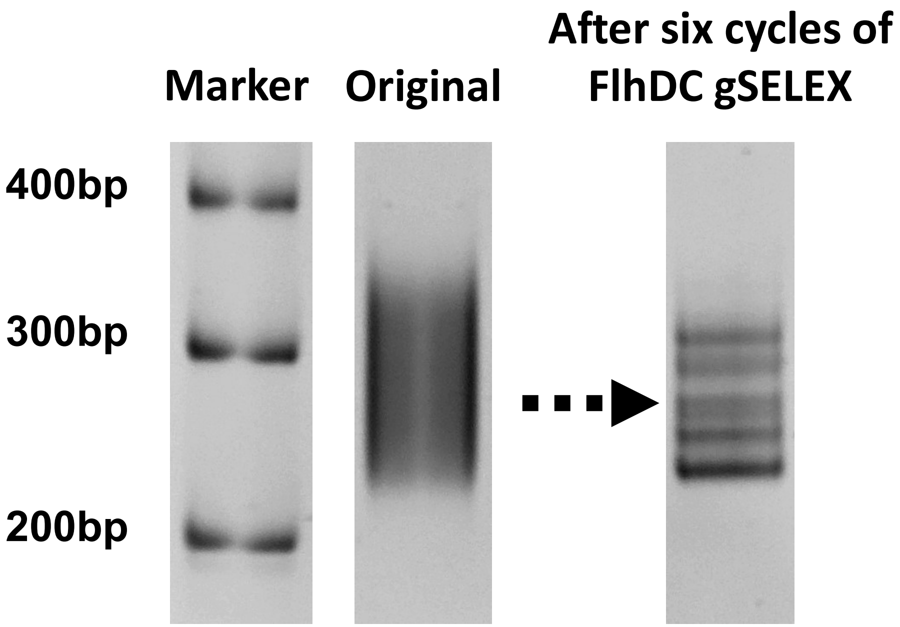

Figure S1. DNA band patterns of genomic DNA fragments. The SELEX cycle was repeated six times to enrich the FlhDC-binding sequences. The original DNA in gSELEX and the DNA after six cycles were each run on PAGE, and the DNA was stained with GelRed for observation.

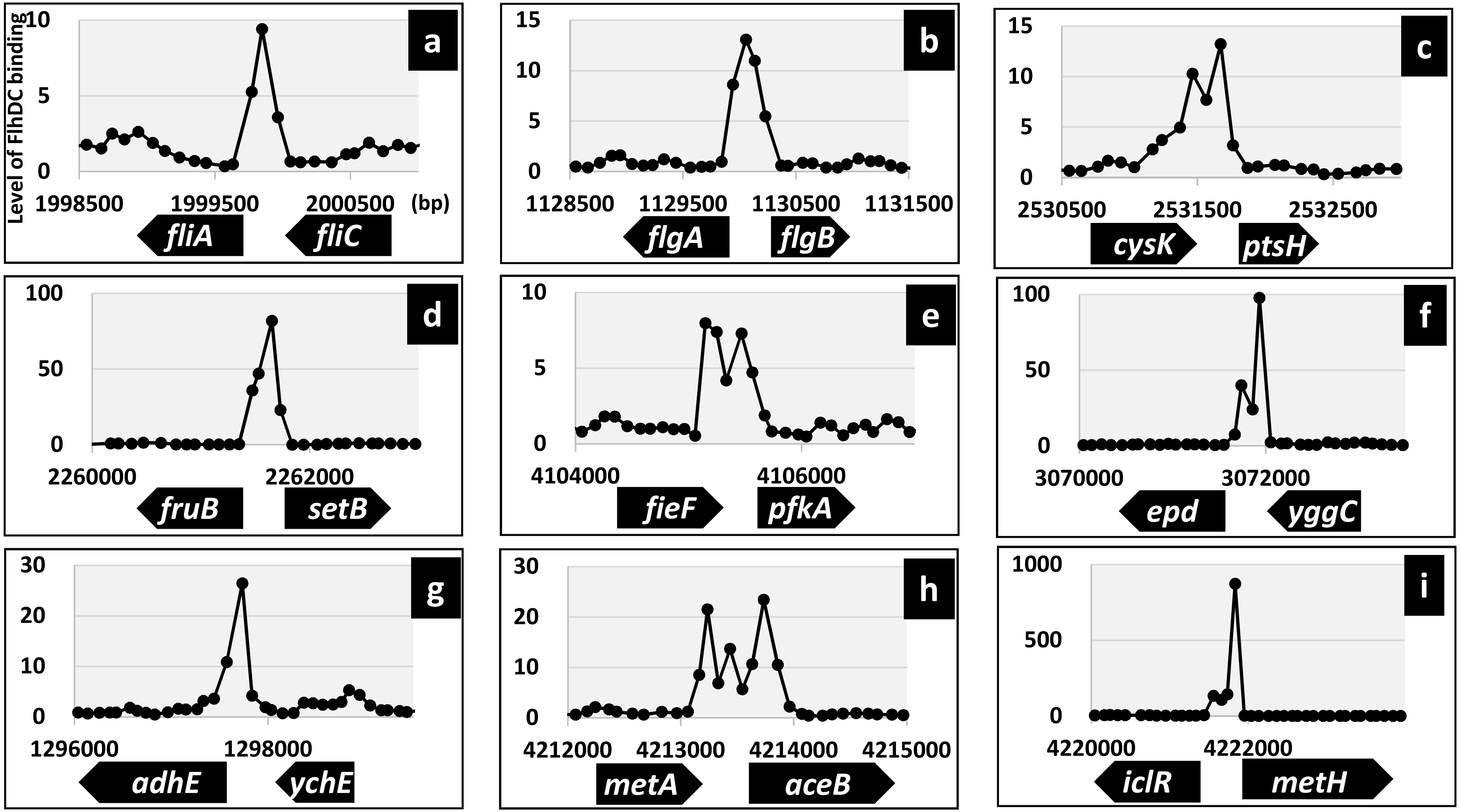

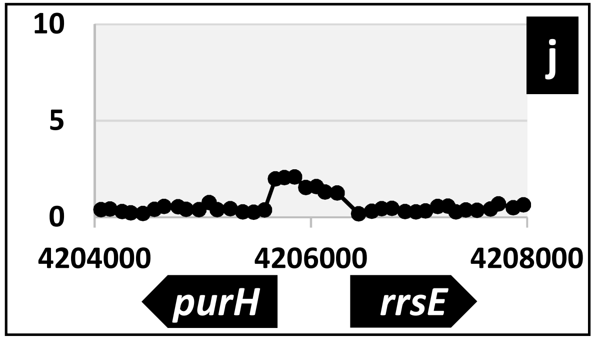

Figure S2. Expanded peaks of the FlhDC-binding sites along the *Escherichia coli* K-12 genome. The peak regions with the binding activity of purified FlhDC complex protein were expanded. Because gSELEX utilized genomic DNA fragments of 200-300 bp, the DNA fragments enriched by binding to FlhDC were detected as peaks consisting of multiple consecutive probes in the tiling array used here with a resolution of approximately 100 bp intervals. (a, b) FlhDC known target genes involved in flagellar formation. (c-i) FlhDC newly target genes involved in sugar utilization and carbon source metabolism. (j) Negative control in areas where FlhDC binding was not observed.
